# Radiographic evaluation of dental age of adults using Kvaal’s method

**DOI:** 10.4103/0974-2948.71053

**Published:** 2010

**Authors:** Ridhima Sharma, Anurag Srivastava

**Affiliations:** *Department of Oral Medicine and Radiology, Government Dental College and Hospital, Jaipur, India*

**Keywords:** Age estimation, Kvaal’s method, RVG

## Abstract

**Introduction:**

It is a well-known fact that the assessment of the dental development can be related to an individual’s age, but after the age of 21 years when the wisdom teeth also complete their development, there arises a need for an optimal age estimation procedure. With advancing age, there is a reduction in the size of the pulp due to secondary dentin deposition and a measurement of this reduction can also be used as a parameter to assess the age of the individuals, both in the living and dead.

**Aims and Objectives:**

The purpose of the present study was to evaluate the feasibility of this approach in the estimation of age of adults, using Kvaal’s method in the set sample.

**Materials and Methods:**

The material consisted of the digital long-cone intraoral periapical radiographs from 50 subjects of either sex in the age group of 15–60 years, who were selected after evaluation for the set inclusion and exclusion criteria. The pulp width and length from radiographs of 6 selected teeth, namely, maxillary central incisor, lateral incisor, and second premolar and mandibular lateral incisor, canine, and first premolar of either right or left side were measured using the RVG trophy software [Trophy® Windows is a software program supplied by Trophy Radiologie (Trophy Windows Version 5.03, Copyright 1993-2002,Trophy RVG patented by Trophy, Chicago)]. In order to compensate for the differences in magnification and angulation, various ratios were calculated and the mean of all ratios (M) was taken as the first predictor, while the difference between the mean of 2 width ratios and the mean of 2 length ratios (W – L) was taken as the second predictor. Different regression formulae for all 6 teeth, 3 maxillary teeth, 3 mandibular teeth, and each of the individual teeth were derived and the age was assessed. The assessed age was then co-related with the actual age of the patient using the Student’s *t* test.

**Results:**

The results showed that the coefficient of determination (R^2^) was the strongest (0.198) for the mandibular first premolar indicating that age can be estimated better with this particular tooth. No significant difference was observed between the estimated age and the actual age for all (*P*>0.05) except in mandibular lateral incisor and maxillary lateral incisor, where a significant difference was observed.

**Conclusion:**

To conclude, the results of the present study suggest the feasibility of Kvaal’s method for age estimation in the set sample

## Introduction

Forensic odontology is one of the most unexplored and intriguing branches of forensic sciences. Age estimation constitutes an important factor in the identification of an individual in forensic odontology and search for optimal age estimation procedures has continued over the years until the present day.[1]

Various dental age calculation methods are described in the literature, but most of them offer a destructive approach in the form of extraction and preparation of microscopic sections of teeth, which may not be acceptable for ethical, religious, or scientific reasons.[2] Any method used for age estimation in forensic sciences should clarify issues with significant legal and social ramifications for individuals as well as for the community.[3] In such circumstances, a radiographic approach if used, offers a relatively nondestructive method and eliminates the need for extraction of teeth.[2]

The dental pulp is a delicate soft tissue enclosed within the confines of calcified structures, namely, dentin and enamel, and is well protected from the external tooth environment. The regressive changes in the pulp have also been related to age. It is a well-known fact that both, the developmental and regressive changes to the tooth can be related to chronological age.[4] The size of the pulp decreases with age due to the deposition of the secondary dentin, and this is a continuous process that occurs throughout life.[5]

Hence dental pulp can be used as a parameter to assess the age of an individual even during later periods of life, when other methods cannot be employed. Kvaal’s method[2] is one such method, which was initially applied to intraoral periapical radiographs and very recently on digital orthopantomographs (OPGs) for estimating the age of an individual.[6]

## Materials and Methods

The present study comprised 50 subjects of either sex in the age group of 15–60 years from whom informed consent was obtained after explaining the aims of the study and the procedure in the language understandable to them. For each subject, a thorough medical history was elicited to rule out any kind of systemic disorders and simultaneously a proof of their date of birth, preferably in the form of a copy of their birth certificate, was obtained and submitted to another observer who was not associated with the procedure. Those subjects who failed to produce their authenticated proof of date of birth were excluded from the study.

Digital intraoral periapical radiographs were acquired using Trophy RVG machine with the exposure factors of 65 KVp and 8 mA for 0.2 s for the 6 teeth of either right or left side, i.e., the maxillary central incisor, maxillary lateral incisor, maxillary second premolar, mandibular lateral incisor, mandibular canine, and mandibular first premolar. The subjects in whom the required teeth were missing/impacted/carious/filled/prosthetically restored/malposed/had periapical or pulpal pathologies, or morphological abnormalities, including attrition/abrasion/ erosion were not taken into consideration.

For each of these teeth, the following measurements were made using the RVG trophy software:

1. maximum tooth length
2. pulp length
3. root length on mesial side
4. pulp width at level a (cementoenamel junction [CEJ]), level c (midroot level), and level b (midpoint of c and a)
5. root width at level a (CEJ), level c (midroot level), and level b (midpoint of c and a)

In order to reduce the possible effects of variation in magnification and angulations of the radiographs, the following ratios were calculated:

1. Root length/tooth length (*T*)
2. Pulp length/tooth length (*R*)
3. Pulp length/root length (*P*)
4. Pulp width/root width at level a (*A*)
5. Pulp width/root width at level b (*B*)
6. Pulp width/root width at level c (*C*)
7. Mean values of all ratios (*M*)
8. Mean value of width ratios from levels b and c (*W*)
9. Mean value of length ratios *P* and *R* (*L*)
10. Difference between *W* and *L* (*W – L*)

Age was assessed for all the subjects by regression using 2 predictors, where the mean of all ratios (*M*) was taken as the first predictor, while the difference between the mean of the 2 width ratios and the mean of the 2 length ratios (*W – L*) was taken as the second predictor. Prior to running the regression, correlation was carried out to find the relationship between the age and the variables. Different regression formulae for all the 6 teeth, 3 maxillary teeth only, 3 mandibular teeth only, and each individual tooth were derived and the age was assessed for each individual. The entire statistical analysis was performed using the SPSS (Version 13) software. The assessed age was then compared with the actual age of the patient using the Student’s *t* test.

## Results

The study comprised 21 males and 29 females. The mean age of the subjects was 25.78 years for males and 22.73 years for females. Correlation between age and the ratios of measurement from each tooth is depicted in Table 1. It was seen that there was a significant co-relation between age and “*M*”for upper second premolar and lower first premolar. A significant co-relation was also seen between age and the second predictor “*W – L*” using the upper central incisor.

**Table 1:** Correlation between age and the ratios of measurement

	Upper central incisor	Upper lateral incisor	Upper second premolar	Lower lateral incisor	Lower canine	Lower first premolar	
P	−0.09	−0.06	−0.15*	−0.12	−0.34	−0.44*	
T	−0.29	0.05	−0.05	−0.07	−0.30	−0.38*	
R	0.13	−0.11	−0.12	−0.07	−0.05	−0.23	
A	−0.17	−0.32*	−0.13	−0.17	−0.11	−0.11	
B	−0.32*	−0.22	−0.14	−0.28	−0.20	−0.21	
C	−0.36*	−0.36*	−0.24	−0.29	−0.25	−0.25	
M	−0.28	−0.24	−0.17*	−0.27	−0.35	−0.43*	
W	−0.34*	−0.30	−0.19	−0.29	−0.23	−0.24	
L	−0.01	−0.08	−0.14	−0.11	−0.23	−0.37*	
W-L	−0.36*	−0.12	−0.04	−0.22	−0.04	0.19	

* denotes significant correlation

The regression equations derived for assessing the age are depicted in Table 2. It was observed that when the selected 6 teeth were taken individually, the coefficient of determination *R*^2^ was the strongest for the lower first premolar indicating that the age can be estimated better with this particular tooth when “*M*” and “*W – L*” are considered as predictors of age. Only ”*M*” was found to be a significant predictor (*P*<0.05) in this case.

**Table 2:** Regression analysis

Teeth	Regression equation	R^2^ (coefficient of determination)	Upper second premolar
All 6 teeth	Age=55.9 − 52.6 (M)	0.072	M
	− 6.87 (W–L)		
Lower canine	Age=113 − 107 (M)	0.151	M
	+ 21.4 (W–L)		
Lower lateral	Age=51.8 − 49.4 (M)	0.078	None
incisor	− 9.4 (W–L)		
Lower first	Age=91.4 − 82.3 (M)	0.198	M
premolar	+ 16.5 (W–L)		
Upper second	Age=37.3 − 25.0 (M)	0.031	None
premolar	− 5.5 (W–L)		
Upper lateral	Age=69.9 − 91.5 (M)	0.084	None
incisor	− 25.0 (W–L)		
Upper central	Age=20.9 − 61.2 (M)	0.183	None
incisor	− 65.3 (W–L)		
Upper 3 teeth	Age=13.2 − 2.25 M	0.020	None
	− 17.9 (W–L)		
Lower 3 teeth	Age=29.42 − 5.89 M	0.011	None
	+ 2.8 (W–L)		

When 3 upper and 3 lower teeth were taken together, it was observed that *R*^2^ was higher for the upper teeth compared with the lower teeth. In the upper teeth as well as the lower teeth, “*M*” and “*W – L*” were found to be insignificant.

When all the 6 teeth were taken together and the age was estimated with “*M*” and “*W – L*” as the predictors, it was found that only “*M*” was a significant predictor and the co-efficient of determination was low.

The age of the subjects was then estimated by substituting the values of “*M*” and “*W – L*” in the regression equation using each individual tooth, upper 3 teeth together, lower 3 teeth together, and for all 6 of them combined, and this estimated age was compared with the actual age using Student’s *t* test [Table 3].

**Figure 1:**
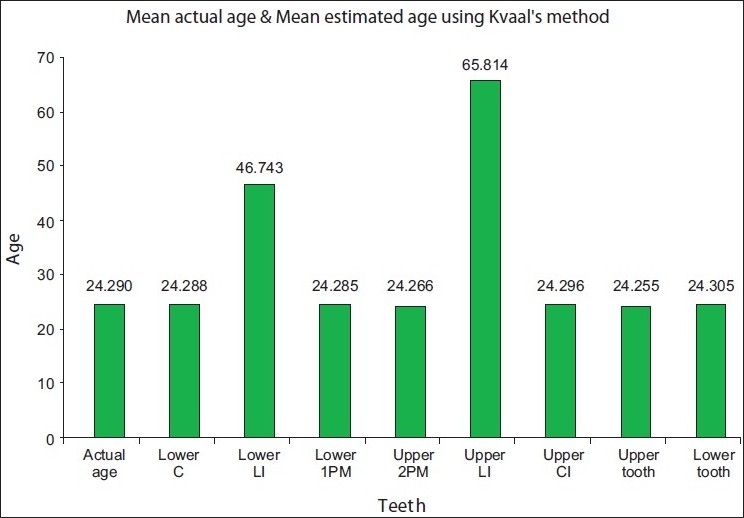
Graphical representation of mean actual age and mean estimated age

From the comparison of actual age and assessed age it was observed that there was no significant difference observed between the estimated age and the actual age for all (*P*>0.05) except in mandibular lateral incisor and maxillary lateral incisor. The bar diagram depicting the comparison between the mean actual age and the mean estimated age is shown in figure 1.

**Table 3:** Comparison of estimated age with the actual age

Tooth	Actual age (mean±SD)	Estimated age (mean±SD)	Mean difference	*T*	*P* value
All 6 teeth	24.29±11.83	24.34±3.15	−0.046	−0.060	0.953
Lower canine	24.29±11.83	24.29±4.58	0.010	0.000	0.998
Lower lateral incisor	24.29±11.83	46.74±5.42	−22.453	−11.050	<0.001*
Lower first premolar	24.29±11.83	24.29±5.27	0.010	0.000	0.998
Upper second premolar	24.29±11.83	24.27±2.08	0.025	0.010	0.990
Upper lateral incisor	24.29±11.83	65.81±5.57	−41.524	−20.340	<0.001*
Upper central incisor	24.29±11.83	24.30±5.06	−0.006	−0.010	0.998
Upper 3 teeth	24.29±11.83	24.25±1.64	0.035	0.030	0.974
Lower 3 teeth	24.29±11.83	24.30±1.23	−0.014	−0.010	0.989

* denotes a significant difference

## Discussion

Based on the study on age estimation of adults from the measurements of pulp size on intraoral periapical radiographs done by Kvaal *et al*., we assessed the age of the subjects using digital long-cone intraoral periapical radiographs of the 6 selected teeth. All the required measurements were made using the inbuilt trophy digital software of the RVG unit. A digital intraoral periapical radiograph in contrast to other radiographs, such as an OPG provides a good image detail and definition without any superimpositions. It was also suggested by Willems *et al*. that it might be worthwhile to produce a calibrated digital image of the radiograph in order to be able to perform digital linear measurements, which might produce the most accurate measurements.[1]

The results of the study by Kvaal showed that the coefficient of determination was highest when the ratios of all the 6 teeth were taken and lowest when mandibular canines alone were taken.[2] Whereas in the present study, it was seen that the coefficient of determination was the highest when lower first premolar was used and lowest when lower 3 teeth were used together.

When the age of the subjects was estimated by substituting the values of “*M*” and “*W – L*” in the derived regression equations and compared with the actual age, it was seen that there was no significant difference between the mean actual age and the mean estimated age in the lower first premolar, lower canine, upper second premolar, upper central incisor, 3 upper teeth taken together, 3 lower teeth taken together, and all the 6 teeth taken together (*P*>0.05), which is in consistence with Kvaal’s study.[2] But a significant difference was observed in the actual and estimated age when the upper lateral incisor and the lower lateral incisor were used.

The difference in the observations in Kvaal’s study can be attributed to the use of a different technique for obtaining measurements. The required length measurements in Kvaal’s study were obtained on conventional radiographs by using vernier calipers and the width measurements using a stereomicroscope with a measuring eyepiece to the nearest 0.1 mm. But in our study, digital radiographs were acquired to obtain the measurements using a standardization procedure.

A similar study was also carried out on digital OPGs of Caucasian population by Bosmans *et al*. in 2005.[6] In their study, they found no significant difference between the actual age and the calculated age based on regression equation of all 6 teeth taken together and for mandibular 3 teeth taken together, which is quite consistent with the present study. They found a significant difference in the actual age and the calculated age for the 6 teeth taken individually and for the upper 3 teeth taken together, but in our study, even the upper 3 teeth when taken together and all the 6 teeth taken together gave no significant difference between the actual and the estimated age.[6] From their study, they concluded that all 6 teeth when taken together were the strongest predictors for age estimation but according to the results of the present study, the lower first premolars were found to be the strongest predictors.

Another reason for the difference in the results of this study from other similar studies can be attributed to the variation in set of sample, which was a set of Norwegian population in the reference study.

Many other studies based on similar parameters have also been carried out and one among them is based on exploring if measurements of the size of the pulp cavity performed on digital OPGs can be used for individual age estimation. In a study, carried out by Paewinsky *et al*. the measurements were made digitally for 6 types of teeth from OPGs of individuals aged between 14 and 81 years. The width ratios of the pulp cavity showed significant correlation to the chronological age and the coefficient of determination (*r*^2^) was highest in the upper lateral incisors (*r*^2^ =0.913) when an exponential or a logistic regression model was constructed. At the same distance with a linear regression model, the coefficient of determination (*r*^2^) reached 0.839.[7]

On similar grounds, Roberto Cameriere *et al*, in 2007, carried out a study to examine the application of the pulp/tooth area ratio by digital periapical images of upper and lower canines as an indicator of age. Separate linear regression equations were obtained for age estimation using upper and lower canines. A variation of 86% with a residual standard error of about 5.4 years was estimated between chronological and actual age and it was concluded that canines can serve as appropriate variables to predict the age of an individual.[8]

The application of RVG was made use of, in a study conducted by Velmurugan, *et al*. in 2008, to determine morphological measurements of the pulp chamber and also to establish the relationship of the CEJ to the roof of the pulp chamber of the maxillary first molars in an Indian population. The results of these measurements revealed that the morphological measurements of the maxillary first molars in the Indian population were similar to those reported by previous studies; the roof of the pulp chamber was found at the CEJ in 96% of the specimens.[9]

Hence, it is quite clear that various studies have come up using digital systems, either in the form of RVG for intraoral periapical radiographs or digital OPGs, to assess the relationship of various tooth parameters with the age of an individual. Accuracy and precision are important in assessing age. Accuracy refers to the closeness of a computed value to its true value. Any difference found can be attributed to many variables, including precision of the method, age distribution of the sample, sample size, and the statistical approach used.[10]

The present study made use of digital intraoral periapical radiographs for estimation of age applying the Kvaal technique and although there are observed variations in the results of different similar studies, yet the feasibility of the technique is certain.

## Conclusion

To conclude, this study was an attempt to apply Kvaal’s method on digital intraoral periapical radiographs to assess the age of individuals in the set sample and the results suggest that Kvaal’s method can be used for age estimation. Furthermore, from among all the chosen teeth, the results may be better when lower first premolar is taken. Also it gives a scope for future studies on larger sample size with adequate representation of samples from different age groups and sex distribution.
